# Hepatic Proteins and Inflammatory Markers in Rheumatoid Arthritis Patients

**Published:** 2017-08

**Authors:** Manel BEN-HADJ-MOHAMED, Souhir KHELIL, Mokhles BEN DBIBIS, Latifa KHLIFI, Hend CHAHED, Salima FERCHICHI, Elyes BOUAJINA, Abdelhédi MILED

**Affiliations:** Biochemistry Laboratory, Farhat Hached Hospital, Sousse, Tunisia

**Keywords:** Hepatic proteins, Inflammation, Iron metabolism, Rheumatoid arthritis

## Abstract

**Background:**

Rheumatoid arthritis is an autoimmune inflammatory rheumatic disease that causes chronic synovial inflammation eventually leading to joint destruction and disability. The aim of this study was to determine the variations of hepatic proteins, myeloperoxidase, and iron in rheumatoid arthritis Tunisian patients and their implications in inflammation and in iron metabolism.

**Methods:**

Overall, 172 patients from the Rheumatology Department of the University Hospital “Farhat Hached”, Sousse-Tunisia between 2011 and 2012, with rheumatoid arthritis (97.1% women, average age: 48±13 yr) and 147 healthy volunteers (70.1% women, average age: 46± 7 yr) were included in this study. Serum hepatic proteins (high-sensitive C-reactive protein, ceruloplasmin, albumin, transferrin, α-1-acid glycoprotein and haptoglobin) were assessed by immunoturbidimetry (COBAS INTEGRA 400, Roche) and ferritin was measured by a microparticulate immunoenzymatic technic (AxSYM, ABBOTT, Germany), Plasma myeloperoxidase was determined by Enzyme-Linked Immunosorbent Assay. Serum iron was measured according to a colorimetric method at 595 nm (CX9-BECKMANN Coulter-Fuller-Ton, CA).

**Results:**

Significantly higher levels of high-sensitive C-reactive protein, α-1-acid glycoprotein, Haptoglobin and myeloperoxidase in patients compared to controls (*P*<10^−3^). Albumin and iron rates were significantly decreased in patients compared to healthy group (*P*=0.026 and *P*<10^−3^, respectively). There were no differences between cases and controls for levels of ceruloplasmin, transferrin and ferritin (*P*=0.782, *P*=0.808, and *P*=0.175, respectively).

**Conclusion:**

The high-sensitive C-reactive protein, α-1-acid glycoprotein, and haptoglobin increased in acute phase proteins in rheumatoid arthritis disease. The pro-inflammatory cytokines affect iron metabolism leading to the iron deficiency and rheumatoid anemia, which influenced Tf and ferritin levels.

## Introduction

Rheumatoid arthritis (RA) is a complex autoimmune disorder with etiology unknown. Several studies have involved both genetic factors (1) and environmental triggers (2). “It is associated with progressive disability, systemic complications, early death, and socioeconomic costs” (3). RA affects approximately 1–2% of the population worldwide (4). As a systemic inflammatory disease, patients with RA have 60% increase risk for cardiovascular disease compared with the general population. “Irrespective of traditional cardiovascular risk factors, systemic inflammatory mediator characteristics of RA are primarily involved” (5). In fact, RA is a chronic inflammatory disease that affects synovial joints. The inflamed synovium is infiltrated by activated neutrophils, macrophages and lymphocytes (T and B cells) extravasated from blood vessels, leads to increased secretion of proinflammatory mediators (6,7), together with expression of adhesion molecules, matrix metalloproteases (MMPs), and hyperproliferation of synovial fibroblasts (7). Persistent inflammation results in the destruction of cartilage and bone. This occurs through oxidative stress, among the other mechanisms, resulting from neutrophil generated ROS including superoxide anion (O_2_^-^, hydrogen peroxide (H_2_O_2_), hypochlorous acids, and possibly hydroxyl radical (OH°) (8). Antibacterial hypochlorous acid and free radical generation will be catalyzed by the hem myeloperoxidase (MPO) (9). The MPO is highly toxic and releases extracellularly upon neutrophil stimulation, it can be detected in serum and plasma of healthy individuals and the concentrations are increased in different diseases (10) and especially in RA (11). Under physiological conditions, the MPO can be binding by ceruloplasmin (Cp) which inhibits its peroxidase activity (12). Serum concentration Cp, called also α_2_-globulin, increases in the inflammation process or infection and this is mostly due to the Cp production in hepatocytes stimulated by proinflammatory interleukins, such as Il-1 and Il-6 (13).

Not only Cp levels increase in inflammation, but also its ferroxidase activity (14) by catalyzing Fe^2+^ to Fe^3+^. Cp, this hepatic protein, seems to play a role in iron metabolism and protect the body from catalytically active Fe^2+^ (15). During the acute-phase reaction, which is often chronic in RA, the proinflammatory cytokines affect iron metabolism, most notably plasma iron levels and the production of transferrin (Tf), ferritin, and hepcidin (16).

We aimed to assess hepatic proteins in rheumatoid arthritis patient’s: inflammation markers (high-sensitive C-reactive protein (CRP_hs_), haptoglobin (Hp)), and those implicated in iron metabolism (ferritin, Albumin (Alb), Cp, α-1-acid glycoprotein (AAG) and Tf) and MPO.

## Materials and Methods

### Study population

One hundred seventy-two RA patients (97.1% women, average age: 48±13 yr) who fulfilled 1987 the American College of Rheumatology Association criteria for RA were included in this study (17). Patients were recruited from the Rheumatology Department of the University Hospital “Farhat Hached”, Sousse-Tunisia between 2011 and 2012. 79.6% of patients have been receiving Methotrexate (MTX), 83.5% have been receiving Prednisone (Pred) and 66.5% have been receiving a combination of MTX and Pred for at least three months. Standard demographic details were collected; including disease duration, Rheumatoid factor (RF), Anti-CCP antibodies, Anti-Nuclear Antibodies and Disease activity score (DAS_28). Patients with DAS-28>3.2 were defined as having high disease activity (HDA) and those with DAS-28≤3.2 low disease activity (LDA) (11).

Patients suffering from chronic disorders such as thyroid dysfunction, liver or kidney disease, inflammatory disease, infection and those who are smoking, consuming alcohol, any antioxidant supplements or biologic therapy at the start of the study or in the preceding month were excluded.

One hundred forty-seven unrelated healthy volunteers (70.1% women, average age: 46± 7 yr), matched for age and ethnicity of the patients, were chosen as the control group.

Informed consent was obtained from all subjects. The National Medical and Research Ethic Committee approved the study protocol.

### Samples and biochemical assays

Ten milliliters of fasting venous blood were collected from each subject in three tubes (sodium fluoride and potassium oxalate and Ethylenedia-mine Tetraacetic acid (EDTA) anticoagulant tubes and a dry tube). The blood samples were centrifuged at 1000 gr for 10 min at 4 °C. The upper phases of serum and plasma were carefully reclaimed and transferred into polypropylene tubes and stored at −80 °C until use.

Plasma glucose levels, renal profile (urea, creatinine, uric acid) levels and serum levels of total cholesterol and triglycerides were measured with colorimetric assays (at 510 nm, 340 nm, 530 nm and 520 nm, respectively) using an automated system (Cx5 and Cx9 ProBechman Coulter-Fuller-Ton CA).

The CRPhs assay was performed by immunoturbidimetric on latex particles. Human CRP agglutinated the latex particles coated with monoclonal anti-CRP antibody. The disorder was measured by turbidimetry (COBAS INTEGRA 400, Roche). Cp, Hp, Tf, and AAG were measured by immunoturbidimetric method on serum at 340 nm (COBAS INTEGRA 400, Roche). Ferritin in serum was determined by a microparticulate immunoenzymatic technic (AxSYM, ABBOTT, Germany). Erythrocyte sedimentation rate (ESR) was determined by measuring red blood cell aggregation in automatic analyzer according to a capillary photometry technology (ALIFAX, TEST 1, THL T_3_ module). Hemoglobin (Hb) was determined by spectrophotometric method in a hematology analyzer (BECKMANN COULTER® LH 750, US). Serum iron was measured according to a colorimetric method at 595 nm (CX9- Coulter-Fuller-Ton, CA).

### ELISA

The human MPO ELISA was ready-to-use enzyme-linked immunosorbent assay based on Sandwich principle (BioVendor Research and Diagnostic (Laboratní madecína a.s.)). Plasma samples (100 microliters) were incubated one hour at 25 °C in microtiter wells coated with antibodies recognizing human MPO. Biotinylated tracer antibody would bind to capture MPO. Then, streptavidin-peroxidase conjugate would bind biotinylated tracer antibody and would react one hour with the substrate tetramethylbenzidine. The enzyme reaction was stopped by the addition of oxalic acid. The absorbance at 450 nm was measured with a microplate reader (Metertech, ∑ 960).

### Statistical Analysis

Database management and statistical analyses were performed by the SPSS (Chicago, IL, USA) software, ver. 17. A *P*-value less than 0.05 was considered as statistically significant. To verify the normality, the Kolmogorov-Smirnov’s test was used. When the data presented a Gaussian distribution, parametric tests were used (Student’s test for differentiating averages and Pearson’s correlation coefficient (r)). When the data did not have a Gaussian distribution, non-parametric tests were used (U Man-Whitney’s test for comparing populations and Spearman’s correlation coefficient (Rho). Data for CRP_hs_ and ferritin were expressed as median and inter-quartile range (IQR).

## Results

Demographic and clinical characteristics of RA patients and healthy controls were summarized in Table 1. There were no significant differences between patient and control groups concerning plasma glucose, urea, and uric acid levels. Serum total cholesterol and triglyceride concentrations were statistically elevated in RA cases compared to the controls while creatinine levels decreased in patients.

**Table 1:** Study group characteristics

**Parameters**	**Patients (n=172)**	**Controls (n=147)**	***P***
Women/Men (%)	97.1/2.9	70.1/29.9	0.000
Postmenopausal women (%)	53.9	15.2	0.000
Age (years)	48±13	46±7	0.089
BMI (Kg/m^2^)	26.47±5.49	27.8±3.69	0.079
Mean disease duration (years)	3.24±2.18	-	-
Glucose (mmol/l)	5.26±1.79	5.07 ±0.68	0.26
Urea (mmol/l)	4.80±1.68	4.84±1.46	0.86
Creatinine (μmol/l)	66.82±17.80	74.91±24	0.004
Uric acid (μmol/l)	210.45±91.28	207.55±90.45	0.79
Total cholesterol (mmol/l)	4.83±1.02	4.47±0.86	0.001
Triglyceride (mmol/l)	1.67±0.83	1.21±0.71	<0.001
Anti-CCP antibodies (%)	64.9	-	-
Anti-Nuclear antibodies (%)	21.9	-	-
RF positive (%)	59.3	-	-
DAS_28	5±2.4	-	-
ESR (mm/h)	38.98±26.19	12.69±10	<10^−3^
NSJ	3±2	-	-
NTJ	4±3	-	-
Hb (g/dl)	12.16±1.45	13.2±1.4	<10^−3^
Anemia (%)	41.9	-	-
Iron supplementation (%)	3.1	-	-
MTX (%)	79.6	-	-
Treatment Glucocorticoid (Prednisone) (%)	83.5	-	-
MTX+ Prednisone (%)	66.5	-	-

Anti-CCP antibodies*:* antibodies against cyclic citrullinated peptide, DAS_28: disease activity score, ESR: Erythrocyte sedimentation rates, NSJ: number of swollen joint, NTJ: number of tender joint, Hb: hemoglobin, MTX: methotrexate; mm/h: millimeters per hour

59.3% of patients had positive RF, 64.9% had anti-CCP antibodies and 21.9% had anti-nuclear antibodies. ESR was significantly higher in patients compared to controls; although, Hb was significantly lower in RA patients.

Hepatic protein variations were shown in Table 2. The RA patients presented significantly higher levels of CRP_hs_, AAG, MPO, and Hp than the healthy group. The levels of albumin and iron in serum of patients were significantly decreased compared to the placebo group. There were no differences between RA cases and controls for levels of uric acid, Cp, Tf, and ferritin. Significant positive correlations were found between the serum Cp levels and the values of ESR (r= 0.268; *P*= 0.011), Hp (r=0.249; *P*=0.009) and Tf (r=0.198; *P*=0.038). In the present study positive correlation between Alb and Tf with a coefficient of r=0.397 (*P*<10^−3^) was obtained in the patient group.

**Table 2:** Variation of hepatic proteins, inflammation markers and iron between patients and controls

**Populations**	**Patients (n=172)**	**Controls (n=147)**	***P***
**Parameters**			
Alb (g/l, x±σ)	42.29±7.32	44.26±5.03	0.026
Cp (g/l, x±σ)	0.231±0.112	0.235±0.095	0.782
AAG (g/l, x±σ)	1.230±1.230	0.870±0.182	<10^−3^
Tf (g/l, x±σ)	3.123±0.687	3.104±0.488	0.808
Hp (g/l, x±σ)	2.15±1.1	1.41±0.45	<10^−3^
MPO (ng/ml, x±σ)	4.70±3.29	3.28±1.35	<10^−3^
Iron (μmol/l, x±σ)	11.511±6.224	14.843±5.989	<10^−3^
CRP_hs_(mg/l, median (IQR))	6(IQR 2.12–15.7)	1.42(IQR 0.610–2.65)	<10^−3^
Ferritin (ng/ml, median (IQR))	25 (IQR 11–47)	17 (IQR 9.75–38.75)	0.175

Positive correlations were observed between the AAG and Hp (r=0.624; *P*<10^−3^) and ESR (r= 0.360; *P*<10^−3^) in the RA patient group. Hp was positively correlated with ESR (r=0.468; *P*<10^−3^).

CRP_hs_ presented some significant positive correlations with several parameters: AAG (Rho= 0.670; *P*<10^−3^), Hp (Rho=0.522; *P*<10^−3^), ESR (Rho=0.336; *P*<10^−3^), MPO (Rho=0.285; *P*=2.10^−3^) and ferritin (Rho=0.160; *P*=45.10^−3^) and a negative correlation with Alb (Rho=−0.179; *P*=0.024). Cp, Alb and Tf concentrations can be depending on the pharmacotherapy applied in the RA patients: in fact, patients treated with the MTX have statistically lower serum Cp levels but Alb and Tf have elevated serum levels with MTX (Table 3).

**Table 3:** Plasmatic protein variations between patients with/without MTX treatment (x±σ)

**Proteins**	**Patients with MTX**	**Patients without MTX**	***P***
Cp (g/l)	0.212±0.1	0.381±0.55	0.01
Alb (g/l)	42.86±7.5	39±6.5	0.023
Tf (g/l)	3.17±0.7	2.9±0.6	0.04

## Discussion

RA, a rheumatic disease, is characterized by inflammation of connective tissue involving different organs in a multisystem way. The diagnosis of these disorders and the evaluation of disease activity are based on clinical laboratory assays showing the increase rates of acute phase reactant proteins (18). These findings were confirmed by our study concerning ESR, CRPhs, Hp, and AAG.

Our results showed an increase in plasma MPO protein and this was in agreement with previous studies (11). Plasma MPO protein concentrations were statistically correlated with CRP_hs_ (11). C-reactive protein (CRP) is an acute-phase reactant produced mainly by the liver, with high levels, in response to acute infections and inflammatory process. In these conditions, the serum concentrations of CRP rise rapidly beyond 10 mg/l with a simultaneous elevation of ESR (19), in fact, our findings showed that CRP_hs_ levels increase in patient group and correlate with ESR. This CRP elevation is not stable because CRP has a half-time of 18 to 20 h. The immunoturbidimetry, the high-sensitivity assay technique, used in our study, can detect CRP with a sensitivity range of 0.01 to 10 mg/l and as consequence, quantifies low grades of systemic inflammation, in the absence of overt systemic inflammatory or immunologic disorders (20). CRP_hs_, one of the best indicators of the acute phase response to inflammation, correlated with Hp in our study. High serum Hp levels characterize chronic and acute inflammation. Its first role is the irreversible binding of free oxyhemoglobin in serum. This complex is then removed within minutes by the reticuloendothelial system. Hp may have an immunosuppressive activity (21).

Concerning the Cp, our results showed no differences between RA cases and the control group but several studies reported increases in Cp levels in RA patients (22, 23). This diminution of Cp concentrations in patients might be due to the Cp quantity, binding to MPO and was not assessed. Our finding showed that Cp was positively correlated with ESR, confirmed the results of other investigators (22, 24).

In addition to the increase of serum Cp levels, a decrease of serum Tf levels was reported in patients with RA (23). We did not find differences on Cp and Tf between groups and this might be due to the treatment but these parameters presented a weak correlation in RA cases.

Several physiologic functions of Cp had been proposed: the roles in copper transport (25), bactericidal activity as an acute-phase reactant (26) and iron metabolism and transport (27). Cp permits the incorporation of iron into Tf without the formation of toxic Fe products (28).

Anemia is defined, by WHO, as a hemoglobin level lower than 12 g/dl in women and 13 g/dl in men. Anemia is highly prevalent among RA patients. It is a typical example of anemia of chronic disease and known as rheumatoid anemia (29). The development of rheumatoid anemia is related to the effects of the proinflammatory cytokines TNFα, interferon gamma (IFNγ), interleukin-1 (IL-1), and interleukin-6 (IL-6) (30). RA patients presented another type of anemia: the iron deficiency anemia caused by medications such as MTX, nonsteroidal anti-inflammatory drugs, and glucocorticoids (31). Infectious agents use iron for growth. One of the defense mechanisms against infections consists in activating the metabolic pathways that increase intracellular iron, which decreases serum iron (32).

Our findings showed no differences in serum ferritin and Tf between RA patients and controls, but iron levels were decreased in cases compared to the healthy subjects. In fact, serum iron was low in both rheumatoid anemia and iron deficiency. Tf was a glycoprotein that binds iron. In inflammatory diseases such as RA, Tf levels decrease (16). Ferritin stores iron in an accessible form. A key molecule limits iron-induced oxidizing stress in health and disease (33). The moderate and non-significant elevation of Tf and ferritin in our RA patient group could be due to the oral iron supplementation in some patients. This showed also, that patients presented both rheumatoid anemia and iron deficiency and this confirms the investigation that serum Tf and ferritin are normal or low in the two kinds of anemia (16).

To evaluate the impact of treatment on plasmatic proteins, these proteins were assessed in patients with different drugs. In our study, only Cp, Tf and Alb differed between patients according to the treatment. On the one hand, serum Cp concentrations were lower in MTX-treated patients and this showed the role of MTX to improve inflammation status in these patients. On the other hand, Alb and Tf concentrations were higher in patients treated with MTX. Low serum albumin was used as a marker of inflammation (34). The cytokines produced in inflammation, particularly interleukin-1, increase the production of CRP and reduce serum albumin and transferrin synthesis (35). MTX treatment can reduce cytokine levels and synovial and systemic inflammation (36).

Despite the importance of MTX for RA, new nanocarriers or nanoparticules used as drug-delivery systems have the potential to achieve the treatment goals of rheumatic conditions and they were able to reduce inflammation at lower doses than an MTX (37).

The most important limitation of this study that we have not included RA cases without any treatment or with MTX monotherapy in order to better evaluate the MTX impact on inflammatory markers.

## Conclusion

During the acute-phase reaction, which is often chronic in RA, the CRP_hs_, the AAG, and the Hp increased in rheumatoid arthritis disease. In addition, the pro-inflammatory cytokines affect iron metabolism, most notably plasma iron levels and the production of Tf and ferritin, which leads to the iron deficiency and rheumatoid anemia.

## Ethical considerations

Ethical issues (Including plagiarism, informed consent, misconduct, data fabrication and/or falsification, double publication and/or submission, redundancy, etc.) have been completely observed by the authors.
